# Development of affective learning in dietetics graduates: A qualitative longitudinal study

**DOI:** 10.1111/jhn.12993

**Published:** 2022-02-03

**Authors:** Marie‐Claire O'Shea, Claire Palermo, Gary D. Rogers, Lauren T. Williams

**Affiliations:** ^1^ School of Health Sciences & Social Work Griffith University Southport QLD Australia; ^2^ Monash Centre for Scholarship in Health Education Monash University Clayton VIC Australia; ^3^ School of Medicine Deakin University, Geelong Melbourne VIC Australia; ^4^ Menzies Health Institute of Queensland Griffith University Southport QLD Australia

**Keywords:** dietetics clinical practice, education communicationand education, qualitative study design and analysis

## Abstract

**Background:**

The development of affective learning during healthcare student education is essential for professional practice. Current studies are limited to short‐term studies with medicine and nursing students. Longitudinal studies are emerging; however, the research within allied health students remains scant. The present study investigates the value of simulation‐based learning activities in relation to affective learning among dietetic students.

**Methods:**

A double hermeneutic, interpretative phenomenological approach (IPA) approach was employed, followed by an analysis of the trajectory of participants' affective learning across three‐interview time points via the application of Krathwohl's affective learning levels.

**Results:**

The simulation developed affective learning in four of the six participants, specifically in relation to their view of themselves as practitioners and their understanding of their future responsibilities to patient care. Three key themes were identified in the participants: (1) feeling of workforce readiness, (2) valuing lifelong learning and (3) attitudes towards interprofessional teamwork.

**Conclusions:**

This IPA methodology described dietetic students' affective learning development as they transitioned to practice as graduate health professionals. Simulation‐based learning is one activity that enhances students' learning in the affective domain and educators should consider its value within their programs

## Key points

- Simulation can enhance affective learning among dietetic students.
- The simulation developed affective learning in four of the six participants, specifically in relation to their view of themselves as practitioners and their understanding of their future responsibilities to patient care.
- Three key themes were identified in the participants: (1) feeling of workforce readiness, (2) valuing lifelong learning and (3) attitudes towards interprofessional teamwork.

## Graphical abstract

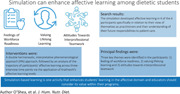

## INTRODUCTION

Affective learning, which focuses on values and attitudes, has long been a key component of health professional education, given the need to work with diverse patient groups in complex situations.^1^, ^2^, ^3^ As one of the three domains of learning, affective learning sits alongside the psychomotor and cognitive domains.^4^ Following the initial description of learning levels in the cognitive domain by Benjamin Bloom in 1956, a model for the affective domain was developed by Krathwohl *et al*.^5^ in 1964. Affective learning has been categorised in a hierarchy of five levels: *receiving*, *responding*, *valuing*, *organization* and *characterization*.^5^

Affective learning processes, related to emotions, attitudes and motivation, also contribute to the development of cognitive and psychomotor domains.^6^ The governing bodies of all health disciplines provide frameworks of competency or entry‐level graduate standards. When reviewing these standards, a varying terminology is used to describe affective learning or related attributes in each profession, with no uniformity evident^2^, ^3^, ^6^, ^7^, ^8^ despite the three domains being ‘extricably intertwined’.^9^, ^10^ In medical education, communication and teamwork have been identified as affective domain skills^3^; however, these cannot be isolated, and other affective aspects of respect, responsibility, duty ^7^ and professionalism^11^ are also at play. Among the few studies in this area with dietetic students to date, affective learning has always been paired with cognitive learning and the studies have not focused on defining each domain separately.^12^, ^13^, ^14^ Aspects of professionalism in dietetics students have been considered difficult to describe^15^ and assess with current knowledge tests in relation to competency‐based education.^16^, ^17^ Despite the widely‐recognised importance of the affective learning domain, measuring or assessing its development in health students has received far less study.^6^, ^11^, ^18^

In dietetics education, the literature investigating affective learning is scant. Of the evidence that exists, work integrated learning experiences with associated blog entries support transformative learning through critical reflection where students create new meaning from their experiences.^13^ There remains a need for evidence of the affective behaviour changes that occur as students develop into practitioners. Although the literature in student healthcare research has recognised the need to measure^8^, ^19^ and assess affective learning^2^, ^3^, ^18^ the range of approaches remains varied and mainly focused on the short term, such as during or immediately after the learning.^11^, ^20^ Longitudinal studies of affective development over time are beginning to emerge but remain rare.

### Teaching and learning approaches to develop affective learning skills

Various teaching techniques are known to enhance affective learning in health professional students. Studies supporting journaling and reflective practice have been documented^1^, ^2^, ^6^, ^19^, ^21^ with various assessment criteria having been established to measure the level of affective development.^11^, ^18^, ^22^ The development of motivational interviewing skills has been shown to increase health student self‐efficacy^23^ and communication skills and have been linked to increased professionalism.^18^ The immersive nature of simulation‐based learning (SBL) offers a safe space for student learning and has been utilised in nursing education to incorporate more depth into affective domain development activities.^2^, ^24^ When combined with interprofessional learning (IPL), simulation can be a ‘powerful affective teaching' method, combining communication and collaboration amongst health professional students.^18^

We would contend that the development of the affective domain is essential to the demonstration of competence in graduate dietitians internationally.

The question remains, how can affective learning be engendered in dietetic students? At one Australian university, SBL activities are embedded throughout the 4‐year undergraduate program, with the majority of SBL completed during the third year of study. These SBL include both uni‐ and IPL activities and include human simulated patients. (Supplementary Table A2). The present study was conducted to investigate the value of SBL in relation to professionally‐focused affective learning among dietetic students. More specifically, the study addressed the research questions: (1) What changes in affective learning are observed in dietetics students as they move from student to graduate practitioner? (2) Can dietetics graduates demonstrate acquisition of affective domain capabilities?

## METHODS

The researchers employed a double hermeneutic, interpretative phenomenological approach (IPA) approach, followed by an analysis of the trajectory of participants' affective learning across time via the application of Krathwohl's affective learning levels^5^ as previously described by Rogers *et al*.^22^ Ethical clearance for this study was obtained (GU2019/009) prior to study commencement.

### Participant recruitment

All students (*n* = 56) enrolled in a communication and counselling course of a dietetics program at an Australian university were provided with an overview of the study and invited to participate. Interested participants expressed their willingness to participate via email and were then provided with the study information sheet and interview times were arranged.

Each participant was interviewed on three separate occasions: the first prior to clinical placement, the second immediately after clinical placement, with the final interview taking place 6 months after graduation. A semi‐structured interview schedule, adapted from the work of Smith and Osborn,^25^ was pilot tested with PhD students and adjusted following feedback, prior to implementation with participants. All interviews were led by one researcher (MCO), a female dietitian involved in the program. Half of the initial interviews were jointly conducted by one other researcher in the team (GDR or CP). All interviews were recorded and transcribed verbatim. Consent to participate in the study and record each interview was obtained in both written and verbal format at the commencement of the first interview(Supplementary Mat_1).

### Statistical analysis

Prior to undertaking any analysis, the relevant researchers met online to reflect and discuss their positioning in regard to the study.^26^ The transcripts were then analysed by two investigators (MCO and CP) and a research assistant independent of this study, applying a method adapted from Rogers *et al*.,^22^ using NVIVO, version 12 (QSR International). A step‐by‐step approach to analysis of the transcripts was undertaken based on the work of Smith *et al*.,^27^ van Manen *et al*.^28^ and Saldaña.^29^ We utilised a highlighting method, where transcripts were viewed as section or phrases, rather than line by line. This ‘double hermeneutic process' is explained simply as ‘the researcher making sense of the participant, who is making sense of x’.^22^, ^27^ In this method, the analyst first looks for how the participant has made sense of their learning experience. Then, as a second phase, the analyst seeks to make sense of the participant's sense‐making in psychological terms, looking for evidence of affective domain learning that may not have been fully evident to the participant at the time. Rather than frequency analysis, which is the number of times a theme could be counted in the analysis, the resulting emergent themes were identified as phenomenon avoiding hierarchical thinking.^30^ In doing so, the analyst aimed to interpret the meaning of the identified phenomenon, or the participants' lived experience.^31^ Interpretations were discussed by the researchers until agreement was achieved. To align more closely with true IPA methodology, the findings were reported with interpretations of meaning rather large sections of direct quotes.^32^, ^33^

To answer the first research question and identify evidence of affective learning and changes across time, the researchers reviewed each participant's transcript series against Krathwohl's five levels of affective learning: *receiving*, *responding*, *valuing*, *organization* and *characterization*.^5^ To answer the second research question, the emergent themes were reviewed and linked to the affective domain where possible. The method previously described by Rogers *et al*.^22^ was employed: (i) identification of examples of affective learning in transcripts, (ii) comparison with the Krathwohl's level of learning^5^ and (iii) identifying the highest Krathwohl level of affective learning for which there was evidence in the transcript. To maintain research rigor, the identified themes and associated student quotes were compared and discussed to identify and acknowledge the lens through which each researcher viewed the transcripts. Minor discrepancies in interpretation were resolved by discussion.

## RESULTS

Seven female third‐year dietetics students initially consented to participate in the sequence of in‐depth interviews for the study. Six students completed all three interviews. One did not and was removed from the study. One participated in interview #2 twice as a result of an extension of clinical placement for remedial purposes.

### IPA and Krathwohl's levels analysis

**Figure 1:**
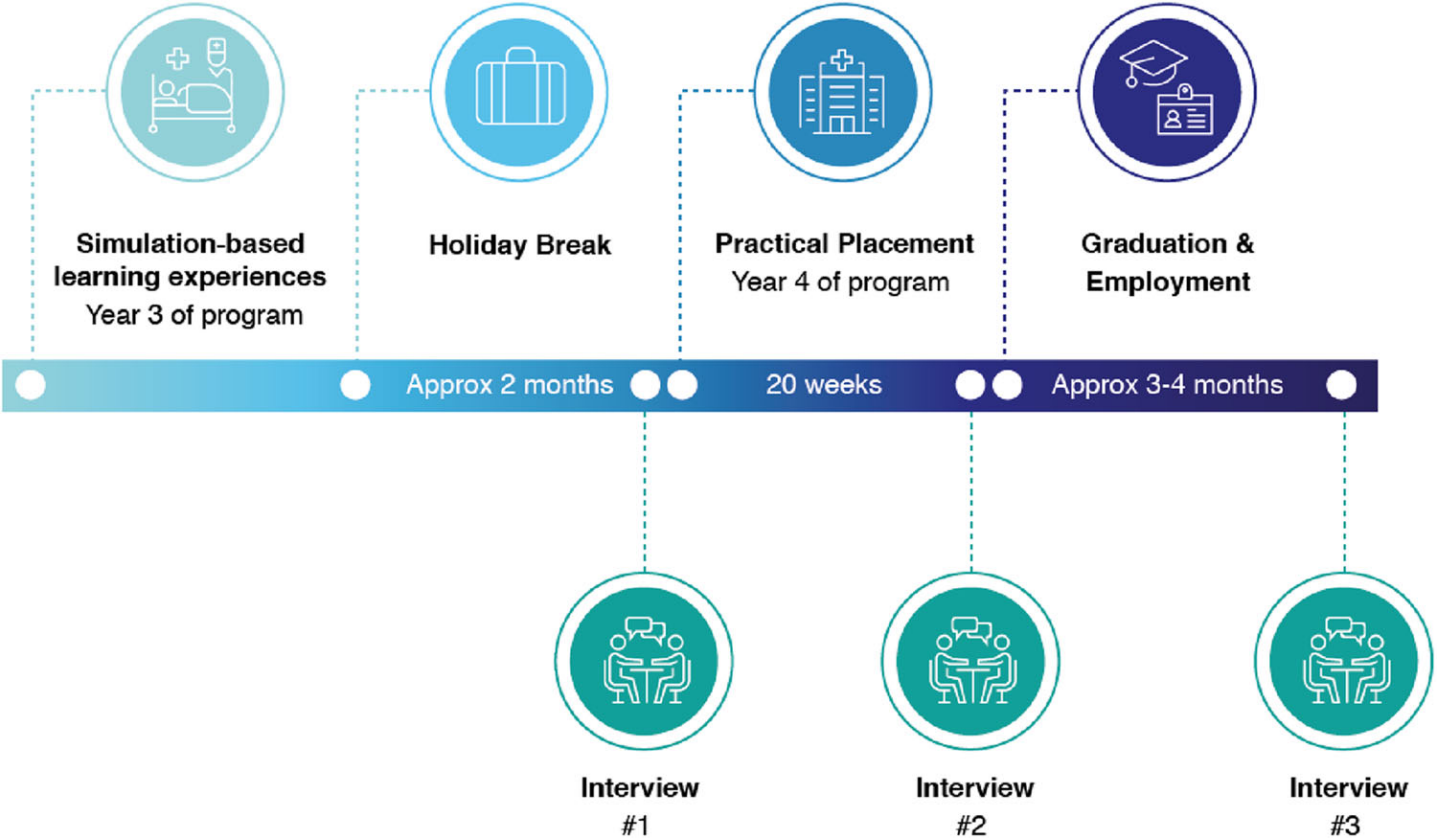
Visual representation of interview timeline in relation to simulation activity within program of study, placement, graduation and employment

The results are presented in two sections. First, evidence of affective learning across the three time‐points is reported and tabulated using Krathwohl's five levels of affective learning^5^ (Table 1). Second, a summary is provided of the development of the attributes of affective learning across the course of the study evident in the participants and categorised into key themes. Quotes and examples have been linked to participant and interview number using ‘P’ to indicate the participant ID (01‐06) (Table 1), followed by a hyphen (‐) and then the interview number (1,2 or 3) (Figure 1).

**Table 1:** Examples of Krathwohl's affective learning levels achieved for each participant^5^

Participant 1 (P01)	Participant 2 (P02)	Participant 3 (P03)	Participant 4 (P04)	Participant 5 (P05)		Participant 6 (P06)
***Receiving***: is being aware of or sensitive to the existence of certain ideas, material, or phenomena and being willing to tolerate them *Evidence* — *the student has at least noticed some aspects of the experience that might ultimately lead to affective learning. For example, a report of something the student found novel or interesting but no discussion of the emotions that the experience has engendered in them*						
**(P01‐1)**: You got to see the other professionals, and you got to see what the doctors do, the pharmacists do. It was really good to be able to see your scope of practice in play	**(P02‐1)**: They didn't know what they were doing in terms of nutrition, and I didn't know what I was doing in terms of medicine. But when we worked together there was no academic difference because we both knew what we were doing on our own paths	**(P03‐1)**: I thought that was the most lifelike because all the occupations were there. Yeah. Because all the occupations are there you could bounce ideas off each other and other people	**(P04‐2)**: I like to work on my own but I also like to know that there's a team around me as well.	**(P05‐1)**: [Simulation has] given me a little insight of what it might be like so that I can interact with other disciplines and be okay talking to other people	**(P06‐1)**: I was afraid of interacting with the real patient or actor. I was very afraid of it. I was very very nervous … I remember just before I entered [the room] ‘he's just a human. Just like the rest of the actors, nobody is going to judge me’	
***Responding***: is committed in some small measure to the ideas, materials, or phenomena involved by actively responding to them *Evidence – the student has reflected on some of the experiences and has identified their own intellectual and, especially emotional reactions to them*						
**(P01‐1)**: I just realised I am not as terrible as I thought I might be, or what I'm going to say isn't as incorrect	**(P02‐1)**: I actually felt like we were important. We felt like we were important and actually achieving something. It wasn't just a simulation and we were just learning. I felt we were all working as a team to treat a real patient	**(P03‐1)**: Then we went back into the room and that's where the med students started firing questions and I could actually answer them. I was like this is nice! I was thinking they'll know all of that for sure but they didn't. So it was nice to be able to teach them something	**(P04‐2)**: Nursing staff l found were always quite busy but they would still stop and give you the time	**(P05‐1)**: I was out of my comfort zone. I didn't have a criteria to meet. That was the learning from me	**(P06‐2)**: I don't think we just take food but I think like, um maybe the other students look at us and think oh what are you doing? But I do think that they learned from us and what we do, because when we had that debrief all together, they had questions and we were able to teach them things they didn't know. So that was really good	
***Valuing***: is willing to be perceived by others as valuing certain ideas, materials, or phenomena *Evidence – the student has gone beyond just recognition of the personal impact of an experience or appreciation that the experience has enabled them to learn something about themselves as a person that they see as valuable or important*						
**(P01‐2)**: I think that I didn't have or didn't value rapport building or those interpersonal skills or active listening I probably wouldn't get very far in patient care	**(P02‐2)**: I'm a rip the band aide off kind of person. I'd rather just get in, get it done and learn from it after. Learn from the experience afterwards	**(P03‐2)**: I don't think there was that nervousness or that stress in talking to them [the doctor] and I think it was once you get the first phone call out of the way it was like oh yeah okay I will call you when I need something and that worked well	**(P04‐3)**: I was probably more surprised by the nurses and how busy they were or appeared to be and yeah just that feeling of them not listening to you	**(P05‐2)**: Never thought I would be that person and you sort of get to a stage where you've learned so much and then all of a sudden I feel like there's no more brain space left, how am I going to continue to learn and keep going but that's the stress and letting yourself get into your head and contemplate everything and start second guessing. And it's that spiral effect. For me that was a big learning curve, identifying my stresses because I thought I had that under wraps. And it's that spiral effect		**(P06‐4)**: I learned that I need to be myself and need to be honest with everything I do. But I don't need to share all my emotions. I just need to choose and pick and show the emotions that I need to show and the emotions that that patient would accept
***Organization***: is to relate the value to those already held and bring it into a harmonious and internally consistent philosophy *Evidence – the experiences have had a significant impact on the student's value system or world view in relation to an aspect of future practice*						
**(P01‐2)**: It's in those moments where you realise you can know it all and have all the skills but if you can't show empathy and if you can't be just a human being and take off the white coat so to speak, um anything you say after that moment where you don't demonstrate empathy, or good rapport building is kind of out the window. You've lost the patient and that's a shame	**(P02‐3)**: The reflections may seem like a pain at the time because they are small assessments and they take time but overall they are teaching you to be a reflective person which is really important for improving yourself professionally and personally	**(P03‐3)**: Initially I got in the habit of being like I'm a new graduate dietitian, and then I'm like no shut up don't tell people you're a new grad dietitian, just tell them you're a dietitian and then they're not even going to blink about the fact that you might be new	**(P04‐3)**: I expect them to be approachable, and I don't know and nice and friendly and open to a new dietitian coming in	**(P05‐3)**: When you're talking to your patient one of the big things I got from placement was that food and drink is like their one thing they enjoy and they can have a an opinion on and going into talk to them in a hospital they sort of don't mind talking to dietitians in hospital. It brings a little bit of quality of life to their stay and it's not always bad news, so I feel like I'm the one practitioner they don't mind seeing. So, I was a big advocate for the patient so not for us as dietitians necessarily but having the opportunity to make them feel a little bit better		**(P06‐4)**: Just trying to give them the best service that you can, and best communication and try to build the best rapport you can and it's up to them if they want to take it, or if they want to leave it
***Characterization***: is to act consistently in accordance with the values he or she has internalised *Evidence – the student's value system in relation to an important area of practice has changed or is in the process of changing their professional behavior*						
**(P01‐2)**: But if you understand that everything is transferable I think you would invest more of your heart into it	NA	**(P03‐3)**: I went from like oh everyone, placement is lovely because everyone is equal and everyone is happy to answer each other's questions and everyone has time for everyone to then like oh no, back out and it's not actually like that and some people think they're more important than others	NA	**(P05‐3)**: I think the sink or swim is what starts and then that gives me the confidence to just go for it and just do it but then once I'm in the swing of things and constantly repeating then that's where I improve and where I grow and get better at what I'm doing		**(P06‐4)**: All these challenges turn me out to the person that I am. So my wisdom to them is if you're challenged, just take it on because in the end that will make you a better dietitian
This participant had highly developed sense of role and identity due to prior experience in the field, so it appears that affective learning occurred much earlier than for others	This participant was not working in the field of dietetics, not by choice, so was unable to move past the negative experiences encountered during placement. It was difficult to deeply discuss topics that might allude to affective learning attributes	This participant appeared overly confident in their own skills from Int 1 prior to placement. The interviews did not clearly demonstrate affective learning. Perhaps the participant has not yet faced challenges or situations where a transformation in learning occurred	There is evidence that this participant now has clear expectations as to how an interprofessional team should operate; open, friendly, listening. Although not a change in their own practice, the views of others and their role in the medical team have been determined by the participant	This participant discusses their own learning journey in regards to their own skill development. They progress to considering learning as whole, not just individual skills. Lifelong learning here demonstrates affective development		This participant completed four interviews, due to a remedial placement. Int 4 was conducted post graduation and suggests a reflective practitioner, no longer worried about failing or nervousness. The tone suggested a practitioner ready to continue their learning journey to provide better patient care

*Note*: Three interviews were conducted and are indicated as Int 1 (interview #1: prior to practical clinical placement), Int 2 (Interview #2: immediately after practical clinical placement) and Int 3 (Interview #3: 6 months after graduation from the Dietetics Program). Participant 6 completed an additional interview: Int4.

Abbreviation: NA, not available.

### Affective learning: Evidence of change over time

Four of the six participants demonstrated evidence of *characterization*, the highest level of Krathwohl's affective learning taxonomy.^5^ One of these students, demonstrated this during interview #2, whereas the others did so during interview #3. The two who did not reach *characterization* level showed evidence of the *organization* level of affective learning during interview #2 but did not demonstrate further affective development during interview #3.

Attainment and demonstration of the highest level of Krathwohl's affective learning levels was clearly identified for participant 1. During interview #1, participant 1 displayed evidence of *receiving* and *responding* as they transitioned from a simple description and observation of their role within an IPL team to recognising an emotional reaction to them; frequent use of the word ‘I’ along with a description of their feelings was evident. During interview #2, the participant appeared to demonstrate progression through the next three levels of *valuing*, *organization* and *characterization* as she described the personal impact of the IPL experience and ‘rapport building’ and reflected on why it was valuable to her. In demonstrating *organization*, the participant shifted from a focus on self to a broader worldview where she values ‘empathy’ rather than her intellectual ability, as in *receiving*. A key characteristic of *characterization* is a sense of consolidation of affective learning, where planned behaviour is evident. This participant spoke of ‘transferrable skills', which represents a profound shift from interview #1 where the observation of skills required and the need to possess a specific skill set was of high importance to her.

Conversely, participant 4 did not demonstrate the same progression of affective learning as participant 1. She moved from lower levels of *receiving* and *responding* in interview #2, where she described experiences that might lead to affective learning but stopped short linking these to personal and impactful examples. Following her practical placement experience, this participant provided evidence that the IPL experiences had a significant impact on her value system when she described her expectation of nurses' and doctors' approach to dietitians in the workplace. However, this participant stopped short of *characterization*, with no foreshadowing of a change to her practice or a planned changed to professional behaviour.

There did not appear to be a link between having worked as a dietitian since graduation and the achievement of *characterization* level. For example, P01 at interview #3 was still looking for work as a dietitian yet demonstrated a value system that was congruent with that of the profession. She had moved beyond a focus on the need for skill development and repetition toward recognising the role of feedback in learning, rather than feedback as criticism; ‘feedback … as if it is a reflection on them personally instead of their practice …’ (P01‐3). On the other hand, P04 who was working as a dietitian at the time of interview #3 did not yet show a change in her principles or ideals regarding professional behaviour. This is not to say that the participant was unprofessional, but rather that there was no evidence of a change of her self‐perceived roles and responsibilities as a practitioner. There was evidence of *organization* level affective learning in P04 where patient care was acknowledged as the most important aspect of practice, but the participant was yet to link this to an over‐arching sense of her responsibility as a clinician in a healthcare system. P04 reflected on her patient‐centred care focus during university SBL conducted prior to clinical practice, compared to the sense of responsibility to the patient she felt after graduation:

‘I just know when I saw the patient … and I had to give the … I didn't do it very well because I didn't really know what I was talking about so I was just saying words without actually portraying what I wanted to say, with care’ (P04‐3)

The shift from concentration on themselves towards a patient‐centered focus demonstrated the development of affective learning over time. Prior to practical placement, participants spoke of lectures, theory and their own feelings of their skill level, ‘it really allowed you to see how much of yourself you could bring to the table’ (P01‐1). Six months later, after 20 weeks of practical placement, a metaphor of a ‘bridge’ was used to describe feelings linking university learning with practical experiences: ‘you think about the expectations of the supervisors … and then you think about what university has taught you and then you've got to make a bridge’ (P01‐2). Finally, a genuine shift from a focus on self to their impact on the patient, away from individual and personal learning experiences, was evident: ‘Someone's condition in front of you. Someone's emotions in front of you. It's all just very different. It's not just textbook stuff’ (P01‐3) (Table 2).

**Table 2:** Examples of participant responses showing linked themes and changes across three time points

Question Area	Interview 1	Interview 2	Interview 3
**Past experience of simulation learning**			
**Examples from interview series**	*Interviewer*: You describe CLEIMS as being the deep end, can you explain why you say that? *P05*: It sort of helped me rely on my knowledge and feel a bit more confident that I do know things that I can just do the stuff	*Interviewer* (responding to P05 comment): At the time you remember thinking this is like CLEIMS? *P05*: we were more confident and we were able to step out from the background and say oh dietitian over here I want to put my 2 cents in… So a similar situation but [I was] a lot more confident	*Interviewer*: Can you think about a time in 3rd year when you felt confident and thought oh yes this practice is helping me? *P05*: CLEIMS again, that was my really big turning point where I felt – cause I sort of had to jump in because my partner got a bit nervous and I had to jump in and do it … And that was my turning point when I went yeah I can do this, I've got this
**Learning outcomes**			
**Examples from interview series**	*Interviewer*: Can you think about the courses that you were doing … which of the activities relate to the simulation activities you've listed here *P02*: By the end of it he [the actor] was like fine. It was, I think I use the communication and counselling course really well in that sim. He started off up here and angry any calm down. By the end of it he left on good terms, with at least me. I think that was a really good example of me using my course work	*Interviewer*: And when in placement did that happen? *P02*: so I sat down with my supervisor… I'm a rip the band aide off kind of person. I'd rather just get in, get it done and learn from it after. Learn from the experience afterwards	*Interviewer*: were there other simulations where you walked away and something happened where it really encouraged and boosted your confidence? … so it was the self‐reflection after the fact plus some feedback? *P02*: the reflections may seem like a pain at the time because they are small assessments and they take time but overall they are teaching you to be a reflective person which is really important for improving yourself professionally and personally
**Impact on future**			
**Examples from interview series**	*Interviewer*: So take the example of the speech pathologist. Can you tell me the story about when you met the speech pathologist and what happened? *P04*: That would have been actually during CLEIMS. But the first time yeah I didn't, I had no idea that we would be working together on the chart or working with their food with regard to the puree and all of that. I didn't know that we would work together that closely	*Interviewer*: [you enjoy working as part of a team]. Why is that? *P04*: I think learning what they did and seeing how the difference they make and then how it all intertwines, especially the speechies I guess cause they would sit with the children and do the what can they eat and then talk to me about what they can eat and can't eat. *Interviewer*: How did that affect your job as a dietitian? *P04*: It was better … I like to work on my own but I also like to know that there's a team around me as well	*Interviewer*: why is that at the forefront of your brain? *P04*: you're always working with all the other disciplines so it was a bit more beneficial knowing how you all interact together
**Summary**			
**Examples from interview series**	*Interviewer*: to summarise in a few words how would you describe your simulation experience to the next third year cohort coming through what would you say? *P03*: it was probably just as, if not more beneficial than sitting in class and finishing off assignments	*Interviewer*: Do you think that you have some advice for our 3rd years that are about to embark on [next trimester]? *P03*: Honestly I reckon I remember that stuff [simulations] more than my assignments	*Interviewer*: If you had to describe the simulation experience, your words of wisdom for 3rd years, what would you say about the simulation experience? *P03*: even though it doesn't have that assessment attached to it, probably put in a bit more focus

*Note*: Interprofessional students provide medical care to an actor patient as part of a week‐long simulation.

Abbreviation: CLEIMS, Clinical Learning through Extended Immersion in Medical Simulation.

### Affective learning: Key themes identified in dietetics students and graduates

Three key themes were identified within the affective learning in the participants: (1) feeling of workforce readiness, (2) valuing lifelong learning and (3) attitudes towards IPL teamwork.

#### Theme 1: Feeling of workforce readiness

The participants appeared to be shaping and developing their own views of themselves as practitioners during the final interview especially. All described a sense of confidence enabling them to feel ready to practice. No longer needing to ‘seek approval’ to complete tasks was described by P02 following practical placement, the participants developed a sense of their own resilience, evident in the final interview where they were ‘accepting that things aren't always going to go amazing’ (P02‐3).

All participants described strong feelings of being ‘practice ready’ during their third and final interview. ‘This is my practice. This is what I'm bringing’ (P05‐3). They no longer viewed themselves as a novice student, but rather as an independent practitioner. This was evident with one student feeling like they were ‘the blind leading the blind’ prior to practical placement, then feeling that their opinion was valued in the clinical setting, to a lifting of the ‘student mentality’ (P02‐3).

#### Theme 2: Valuing lifelong learning

Prior to practical placements, participants reported feeling ‘ready’ and ‘confident’ to embark on the 20‐week learning journey, despite some misgivings; ‘I just hope that I have got that knowledge stored and that I can apply it’ (P05‐1). In the final interviews, participants reflected on their placement journey, recalling positive and negative experiences. Over time, students changed from the view that placement was discrete (beginning and end) to the realisation that a formal learning endpoint was never going to emerge. Participants then appeared to acknowledge and value lifelong long learning; ‘if you're perfect from day one, then you would not need to be learning’ (P03‐3).

#### Theme 3: Attitudes towards interprofessional teamwork

Learning ‘with, from and about’^34^ colleagues in other professions commenced during the simulation experiences at university and continued throughout practical placement. The ‘about’ aspects were particularly evident in the participant stories as they became more aware of the role and contribution other disciplines made to patient care. Equally important was the learning that took place about their own role as a dietitian and a newfound insight ‘of what it might be like so that I can interact with other disciplines’ (P05‐1).

Participants were seen to progress from learning *with*, to learning *from* other disciplines^34^ in the clinical setting. ‘I learned a lot from their approach to each patient’ (P05‐3). Participants moved from assessment of the patient through their own eyes, to an understanding of how others interact with the patient, communicate, and undertake tasks. In the *characterization* stage, there was evidence of the team approach developing where participants describe their ability to ‘bounce ideas’ when ‘the multidisciplinary team works together’ (P03‐3). They reflected on the importance of a developed understanding of each other's role and ‘knowing how you all interact together’ (P05‐3).

The participants also reflected on negative IPL encounters particularly relating to the perception of the doctor–dietitian hierarchy. The IPL simulations at university appeared to break some preconceived ideas some participants held about doctors:

‘We just have this mentality that doctors run hospitals. But they know everything and that everyone else is just underneath them but no, it's nothing like that’ (P03‐1)

These feelings were confirmed during practical placement as the doctors were considered ‘just normal people’ (P03‐2). An interesting change occurred as the participants moved into the workforce. There was a newfound ability to distinguish between the profession and the person. The participants no longer spoke globally about a discipline or role, rather they looked beyond the title and saw the other practitioner as a person, acknowledging that the person may have their own beliefs that are not representative of a whole profession; 'some people think they're more important than others’ (P03‐3).

## DISCUSSION

To our knowledge, this is the first longitudinal study of affective learning in dietetic students and graduates. Using a double hermeneutic IPA approach, the present study demonstrated the development of the affective learning domain as dietetic students transitioned into practicing health professionals. Comparison of the affective learning identified with Krathwohl's hierarchical levels^5^ enabled participants' affective learning trajectories to be seen over time. The IPA approach also identified key themes within the development of affective learning that are consistent with the capabilities required of graduate dietitians. These findings can inform the development and refinement of innovative teaching methodologies such as SBL to enhance the affective development of dietetic students.

The inclusion of SBL within health professional education programs has been shown to have positive impacts on affective development in medicine^22^ and nursing,^23^, ^35^ as well as more broadly.^18^ The present study has demonstrated the link between human–patient SBL in dietetic undergraduate education and affective learning, specifically in relation to their view of themselves as practitioners and their understanding of their future responsibilities to patient care. The theme surrounding workforce readiness was directly attributed to the IPL simulations undertaken in year 3 of the program during both the second and third interviews in the present study. The participants described ‘light bulb’ moments where they were able to recall the specific experiences during practical placement that resembled the IPL simulation the year prior, and the profound impact that the activity had had on their practice. After graduation, similar stories were recalled by participants, suggesting a lasting impact of these activities on their learning and practice. There was evidence of a shift in their value systems towards ‘professional responsibility, interprofessionalism and the primacy of patient or client welfare’, which mirrors the findings in the work of Rogers *et al*.^22^ among medical students. Similarly, the dietetic literature provides evidence of the impact of SBL on placement and workforce readiness. SBL in coursework that mimics the work of a dietitian supports preparation for placement^36^ and enhances their preparedness for placement.^37^ Affective learning should be fundamental to all learning objectives in curriculum and SBL included because it can shape affective development.^18^

The present study poses further questions about the assessment of the affective domain in health professional students. The literature indicates common methods of evaluating affective attributes, including self‐reporting questionnaires and Likert scales,^20^ to the formative assessment of professional role development,^2^ to summative assessments required to determine competence in this domain.^3^ These summative assessments, usually undertaken by a preceptor or supervisor to assess professionalism, are reported as uncomfortable because of their subjectivity.^17^ The challenge is therefore to develop a robust tool for assessing affective development that has been trialled and validated and for which assessors have been trained. The present study has identified one possible method for assessing affective learning development using a hierarchical system, althoughg further testing is still required.

This small‐but‐deep longitudinal study has successfully applied an IPA methodology to describe dietetic students' affective learning development as they transition to practice as graduate health professionals. Larger scale studies will be needed to test this proposed methodology further and advance our understanding of the development of affective learning in health professional education over the course of students' study programs and into the workforce. Educators should consider embedding activities that enhance students' learning in the affective domain at the same time as planning to assess their capability in this complex area, which is vital to patient‐centred care.

## CONFLICT OF INTERESTS

The authors declare that there are no conflict of interests.

## AUTHOR CONTRIBUTIONS

Marie‐Claire O'Shea conducted the studies as part of doctoral research under the supervision of the other authors. Marie‐Claire O'Shea wrote the first draft and all authors contributed to subsequent drafts of the manuscript.

## ETHICAL STATEMENT

Ethical clearance for this study was obtained from Griffith University (GU2019/009) prior to study commencement.

## TRANSPARENCY DECLARATION

The lead author affirms that this manuscript is an honest, accurate and transparent account of the study being reported. The lead author affirms that no important aspects of the study have been omitted and that any discrepancies from the study as planned have been explained.

## Supporting information

Supporting information.

Supporting information.
