# Supplementary material for: Development of affective learning in dietetics graduates: A qualitative longitudinal study
Source: J Hum Nutr Diet. 2022 Feb 3;35(5):872–82. doi: 10.1111/jhn.12993 (PMC9545643; doi:10.1111/jhn.12993)
Supplement: Supplementary file 1 — Supporting information. [file JHN-35-872-s001.pdf]

Consolidated criteria for reporting qualitative research (COREQ): A 32-item checklist for interviews and focus groups

No	Item	Guide questions / description
**DOMAIN 1: RESEARCH TEAM AND REFLEXIVITY**		
Personal Characteristics		
1	Interviewer/facilitator	1. MCO 2. CP 3. GR
2	Credentials	1. PhD Candidate/Dietitian 2. Professor/ Dietitian 3. Professor/Medical Doctor
3	Occupation	1. Academic/Lecturer 2. Academic/Researcher 3. Academic/Researcher
4	Gender	1. Femal 2. Female 3. Male
5	Experience and training	1&2. Highly experienced in interviewing, new to IPA. 3. Highly experienced in IPA interviewing approach
Relationship with participants		
6	Relationship	1. Lecturer for participant's coursework 2. Nil 3. Nil
7	Participant knowledge of the interviewer	1. Completing PhD 2. Nil 3. Nil
8	Interviewer characteristics	1. Gender 1,2&3. Positionality
**DOMAIN 2: STUDY DESIGN**		
Theoretical framework		
9	Methodological orientation and Theory	Double hermeneutic, interpretative phenomenological approach (IPA)
Participant selection		
10	Sampling	Convenience sampling
11	Method of approach	Face-to-face
12	Sample size	7
13	Non-participation	1 dropped out after interview 2 and was not contactable upon graduation from program of study
Setting		
14	Setting of data collection	Interview 1: University Interview 2 &3: University or online
15	Presence of non-participants	Nil
16	Description of sample	Enrolled in a dietetics course at one university, and had completed a series of simulation activities prior to interview 1
Data Collection		
17	Interview guide	A semi-structured interview schedule, adapted from the work of Smith, 2008 was pilot tested with PhD students prior to implementation
18	Repeat interview	Three
19	Audio/visual recording	Audio recording
20	Field notes	Notes were made by interviewer during interview and used to refine next interview
21	Duration	Avg time Interview 1: 23mins, Interview 2: 24mins, Interview 3: 41mins
22	Data saturation	data saturation not applicable to this study
23	Transcripts returned	No
**DOMAIN 3: ANALYSIS AND FINDINGS**		
Data analysis		
24	Number of data coders	2 coded the entire data set, 1 more coded half the data set
25	Description of the coding tree	Did authors provide a description of the coding tree?
26	Derivation of themes	Themes were identiifed after coding
27	Software	NVivo 12
28	Participant checking	Each interview informed the next interview so quotes were used from previous interviews and confirmed by participants
Reporting		
29	Quotations presented	Deidentified quotations were presented to illustrate the themes
30	Data and findings consistent	Yes
31	Clarity of major themes	Yes
32	Clarity of minor themes	No

EQUATOR stands for Enhancing the QUAlity and Transparency Of health Research. It is an international initiative that started in 2008 whose main objective is to improve the reliability and value of scholarly publication of health research through promotion of transparent, complete, and accurate reporting. The Network promotes standards, guidelines and checklists of reporting requirements for various types of studies, from clinical trials and observational studies to reviews and case reports.

The complete checklists and full guidelines are available at http://equator-network.org.
