# Supplementary material for: Development of affective learning in dietetics graduates: A qualitative longitudinal study
Source: J Hum Nutr Diet. 2022 Feb 3;35(5):872–82. doi: 10.1111/jhn.12993 (PMC9545643; doi:10.1111/jhn.12993)
Supplement: Supplementary file 2 — Supporting information. [file JHN-35-872-s002.docx]

**Supplementary Material**

Table A: Summary of simulation activities offered to students within the Dietetics Program at one Australian University from which participants were enrolled during the study

| **SBL Name** | **Student Type** | **Dietetics Year of Study** | **Compulsory in Program** | **Duration** |
| --- | --- | --- | --- | --- |
| CLEIMS 3 | Speech Pathology, Physiotherapy, Medicine Occupational Therapy, Dietetics, Exercise Physiology, Pharmacy | 3^rd^ year of 4yrs | Y | 2 x 6hrs |
| CLEIMS 4 | Psychology, Dietetics, Medicine, Physiotherapy, Pharmacy | 2^nd^ year of 4yrs | Y | 6hrs |
| Palliative Care | Dietetics, Exercise Physiology, Pharmacy, Social Work | 3^rd^ year of 4yrs | Y | 2 x 6hrs |
| ISBAR Nursing | Dietetics, Nursing | 3^rd^ year of 4yrs | N | 2hrs |
| Chronic Disease Management 1 (Diabetes) | Dietetics, Exercise Physiology, Pharmacy | 3^rd^ year of 4yrs | Y | 4hrs |
| Chronic Disease Management 2 (Cardiac) | Dietetics, Exercise Physiology, Pharmacy | 3^rd^ year of 4yrs | N | 4hrs |
| IPL in Aged Care | Exercise Physiology,  Counselling, Dentistry, Social Work, Medicine, Dietetics, Paramedicine, Pharmacy, Psychology | 2^nd^ year of 4yrs | Y | 2hrs |
| Communication Skills Series | Dietetics only | 3^rd^ year of 4yrs | Y | 3x 2hrs |
| IPL Documentation SBL Online | Speech Pathology, Rehabilitation Counselling, Occupational Therapy, Dietetics, Exercise Physiology, Physiotherapy, Social Work, Psychology, Counselling | 3^rd^ year of 4yrs | Y | 3x 1hr |

NB: CLEIMS = Clinical Learning through Extended Immersion in Medical Simulation; interprofessional students provide medical care to an actor patient as part of a week-long simulation.

ISBAR = Introduction, Situation, Background, Assessment, Recommendation; a framework, endorsed by the World Health Organisation, to provide a standardised approach to communication

IPL = Interprofessional learning
